# Primary mediastinal atypical meningioma: Report of a case and literature review

**DOI:** 10.1186/1477-7819-10-17

**Published:** 2012-01-21

**Authors:** Akira Mogi, Junko Hirato, Takayuki Kosaka, Ei Yamaki, Hiroyuki Kuwano

**Affiliations:** 1 Department of General Surgical Science, Gunma University Graduate School of Medicine, 3-39-22, Showa-machi, Maebashi, Gunma 371-8511, Japan; 2 Department of Human Pathology, Gunma University Graduate School of Medicine, 3-39-22, Showa-machi, Maebashi, Gunma 371-8511, Japan

**Keywords:** ectopic meningioma, atypical, mediastinum, surgical treatment

## Abstract

Meningiomas are common neoplasms arising from the central nervous system meninges. On the other hand, primary ectopic meningiomas are extremely rare and usually limited to the head and neck region or to the paravertebral soft tissues. Their occurrence in the mediastinum is even rarer. Until now, only 4 cases of primary mediastinal meningioma have been reported in the literature searched on Medline. Because of its rarity and intriguing pathogenesis, we report here a case of primary mediastinal meningioma that was treated by surgical resection. The clinical features, treatment, pathological findings, and prognosis are analyzed, and the literature on ectopic meningioma is reviewed.

## Background

Meningiomas are one of the most common neoplasms of the central nervous system, and the cell of origin for the meningioma is called the arachnoid cap cell, found on the surface coverings of the brain in the pacchionian granulations. Meningiomas are usually benign and slow-proliferating and account for about 15-20% of all intracranial tumors. On the other hand, primary ectopic meningiomas are exceedingly rare, and they are usually found in the head, neck, or paraspinal soft tissues [1]. Other possible ectopic sites include the foot, lung, skin, and mediastinum [2-4]. A Medline search revealed only 4 cases to date of primary mediastinal meningioma [5-8]. Furthermore, only one case of meningioma arising from the anterior mediastinum has been reported [8].

Here, we report an ectopic atypical meningioma arising from the anterior mediastinum because of its extreme rarity.

## Case presentation

**Figure 1:**
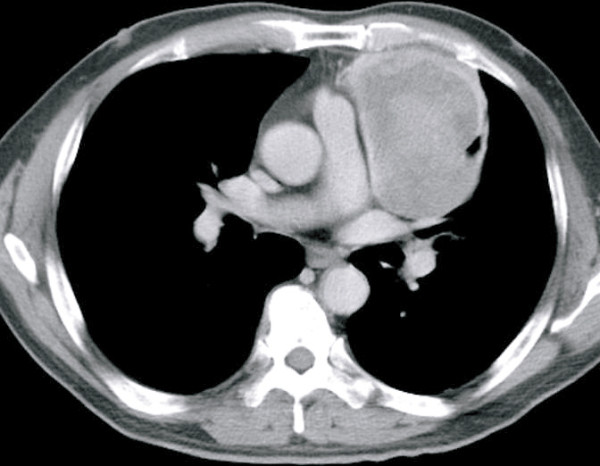
**Enhanced chest computed tomography shows a heterogeneous 9.0 × 7.5 cm mass arising in the anterior mediastinum**.

A 64-year-old male was referred to the Gunma University Hospital with complaints of a progressive oppressive feeling in the left thoracic region. His past medical history was unremarkable. At admission, general conditions were satisfactory, and a physical examination of the thorax was normal. Routine laboratory tests were within normal values, but a chest roentgenogram revealed an oval-shaped anterior mediastinal mass in the left hemithorax. An enhanced computed tomography (CT) scan of the chest revealed the presence of an ovoid mass about 9-cm at its greatest dimension in the left anterior mediastinal region. Its border, adjacent to the left lung parenchyma, was poorly demarcated. The mass showed a heterogeneous densitometric characteristic accompanied with areas of necrosis after contrast enhancement (Figure 1). From the graphical specifications and laboratory studies, an invasive thymoma was strongly suggested, and we decided to perform a radical resection of the tumor.

Written informed consent concerning the operation was obtained from the patient before surgery, and the patient was admitted to the Gunma University Hospital for surgical resection of the tumor. The patient underwent tumor excision and thymectomy through sternotomy with continuous anterior thoracotomy of the third intercostal space. During the operation, the tumor, as seen on the CT scan, was 9-cm wide, elastic, and off-white in color. It was found to have infiltrated the upper lobe of the left lung. Based on this surgical finding, partial resection of the lung together with the tumor was performed with a sufficient surgical margin. On the basis of its morphologic and immunohistochemical features, the tumor was diagnosed as a mediastinal atypical meningioma. The patient had an uneventful recovery and was discharged on postoperative day 6. An ectopic pulmonary site of origin, such as the mediastinum in this case, should be accepted only after the possibility of spread or metastasis from primary intracranial or intraspinal origin has been excluded. One month after operation, the patient had an enhanced magnetic resonance imaging (MRI) of the head and spine, and no abnormal findings were observed, which eliminated the possibility of an intracranial or intraspinal origin. He has been monitored for 12 months as an outpatient without any symptoms of recurrence or metastasis.

**Figure 2:**
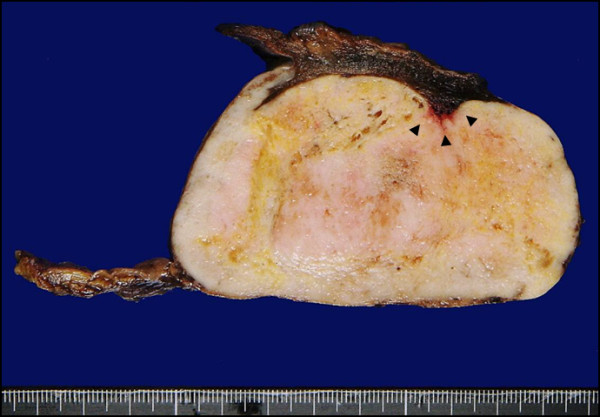
**The oval-shaped tumor had a smooth surface, and its cut surface was homogenously solid with a yellowish-white color**. The tumor was partially involved in the lung parenchyma (arrowhead).

The surgical specimens were routinely fixed with 10% formaldehyde and embedded in paraffin. Tissue sections (6-μm thick) were stained with hematoxylin and eosin (H-E), and periodic acid-Schiff (PAS) with and without diastase digestion. For immunohistochemical studies, tissue sections were incubated using the standard avidin-biotin peroxidase complex (ABC) method with the following antibodies: epithelial membrane antigen (EMA), vimentin, S-100, MIB-1, CD34, D2-40, αSMA, desmin, synaptophysin, and neurofilament. Macroscopically, the resected tumor measured 9-cm in its greatest dimension and adhered to a parenchyma of the left lung. The oval-shaped tumor had a smooth surface, and its cut surface was homogenously solid and yellow-white in color (Figure 2).

**Figure 3:**
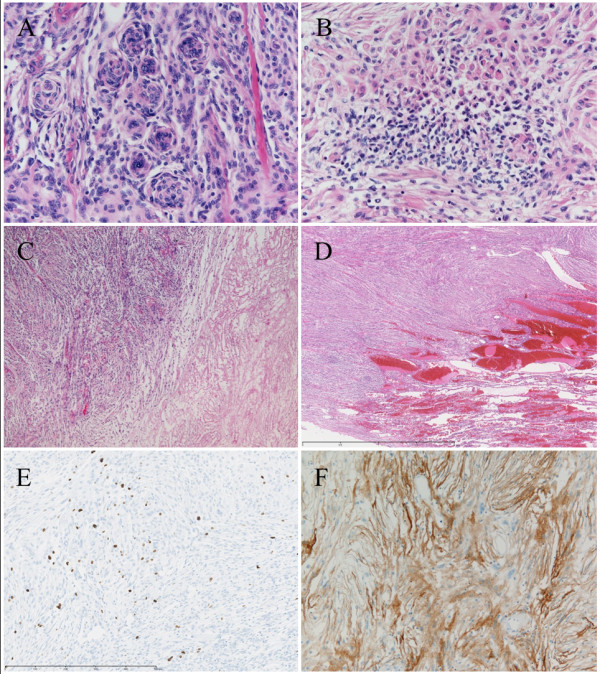
**Numerous typical whorl formations (**A**) and small cells with a high nuclear/cytoplasmic ratio (**B**) were observed in the tumor**. Spontaneous necrosis was occasionally seen (**C**). The tumor was partially involved in the adjacent pulmonary parenchyma (**D**). There were some mitotic figures, and the Ki-67/MIB-1 labeling index was approximately 7% in relatively higher positive-rate fields (**E**). Marked immunoreactivity for epithelial membrane antigen (EMA) was seen (**F**).

Microscopically, the tumor was partially encapsulated and composed of bundles of spindle-shaped cells with elongated nuclei and syncytial nets of tumor cells with oval nuclei. A few tumor cells had an intracellular cytoplasmic inclusion. In some areas, many typical whorl formations for fibrous meningioma were observed (Figure 3A). There were several foci of small cells with a high nuclear/cytoplasmic ratio (Figure 3B). Spontaneous and zonal necroses were occasionally seen (Figure 3C). In addition, the tumor involved adjacent pulmonary parenchyma (Figure 3D). The mitotic index was four per 10 high-power fields (hpf) at the areas where the mitotic figures were most frequently observed, and the Ki-67/MIB-1 labeling index was approximately 7% in relatively higher positive-rate fields (Figure 3E). Immunohistochemical examinations showed positivity to EMA (Figure 3F) and vimentin, focal positivity to S-100, CD34, and D2-40, and negativity to αSMA and desmin. Some oval tumor cells containing large cell bodies were stained for synaptophysin and neurofilament.

## Discussion

Meningiomas usually arise in the cranial cavity or spinal canal. On the other hand, because meningiomas arise from arachnoid cells present in the meninges, they can occur in any location where meninges or ectopic meninges exist. Primary ectopic meningiomas are extremely rare pathological entities and have been reported sporadically. They usually occur in cephalic and paravertebral soft tissues and skin and, more rarely, in the ear [9], temporal bone [9], mandible, foot [2], lung [10], and mediastinum [5-8]. The histopathogenesis of ectopic meningiomas is still unclear. However, three hypotheses are that they arise from an extension of the arachnoid cells along cranial nerve sheaths during development, from the arachnoidal cells trapped in extracranial locations when the skull bone fuses, or from differentiation of Schwann cells into meningocytes [1,11,12].

To make a definite diagnosis of an ectopic meningioma is difficult, and such a diagnosis can only be made on the basis of microscopic morphological and immunohistochemical findings. In this case, and despite the unusual location of the tumor, the morphological and immunohistochemical findings closely resembled a meningioma in the central nervous system. Furthermore, as there was no clinical or radiological evidence of any intracranial or intraspinal lesion, we concluded that the meningioma in this case was of complete mediastinal origin.

In view of the rarity of extracranial meningiomas, caution must be used in ruling out possibilities in the differential diagnosis. Regarding the histological differential diagnosis of epithelial thymomas, they are composed of cells with large numbers of desmosomes often containing numerous cytoplasmic cytokeratin filaments in the desmosomes per se and lying free in the cytoplasm [13]. Cytokeratin filaments are absent in meningiomas. Because of its morphological findings, as typical whorl formation for fibrous meningiomas, solitary fibrous tumors, schwannomas, and hemangiopericytomas could be diagnosed by exclusion. Additionally, hemangiopericytomas could be excluded by positivity of EMA in immunohistochemical results, because EMA stains show typically positive in meningiomas and negative in hemangiopericytomas. Positivity of EMA was compatible with the diagnosis of this tumor as a meningioma. Furthermore, leiomyomas could be excluded by negativity of αSMA in immunohistochemical results.

Histological grading of meningiomas is based on the 2007 WHO classification [14]. Atypical meningioma (WHO grade II) shows increased mitotic activity or three or more of the following histological features: increased cellularity, small cells with a high nuclear/cytoplasmic ratio, prominent nucleoli, uninterrupted patternless or sheet-like growth, and foci of spontaneous or geographic necroses. Increased mitotic activity is defined as four or more mitoses per 10 hpf. In addition, brain invasion was included in an otherwise grade I tumor as an additional criterion for a WHO grade II lesion [14]. In our case, the presence of small cells, spontaneous and zonal necroses, invasion into the pulmonary parenchyma, and mitotic activity of the tumor (four mitotic cells per 10 hpf) correspond histologically to an atypical meningioma. Expression of proliferation markers, such as MIB-1 and Ki-67, has generally shown progressive increases in the labeling index with a WHO grade from 1.00-1.35% for grade I to 1.90-9.30% for grade II or atypical meningiomas and 5.60-19.5% for grade III or anaplastic meningiomas [15,16]. In addition, the median MIB-1 proliferation indices are 3.4% in ordinary meningiomas, 6.6% in atypical meningiomas, and 11.8% in malignant meningiomas [17]. These data further support the classification of the meningioma in this case into an atypical category.

As for the clinical course, the surgical excision was as curative as a complete resection with a sufficient surgical margin. Although this was an atypical meningioma with invasion into lung tissue, no adjuvant therapies, such as radiotherapy, were performed. This decision was based on the fact that Mair et al. retrospectively reviewed the records of 114 consecutive patients with diagnosed WHO grade II meningiomas and reported that radiotherapy for WHO grade II atypical meningiomas was not appropriate after a first-time resection of those lesions in which a gross-total resection has been achieved [18]. In addition, we consulted with the neurosurgeons of Gunma University Hospital, who were experienced in the treatment of cerebral meningioma.

According to our review of the literature, this is the first reported case of primary mediastinal atypical meningioma [Table 1]. Of all the reported cases of mediastinal meningiomas, only one was malignant. All but one was surgically treated alone. The exception was treated with consecutive radiation after surgical resection. Regarding the clinical course of these cases, including this case, every case was disease-free for at least 9 months after surgery except for two cases for which the clinical course was not discussed. Although a longer follow-up is necessary, the findings strongly suggest that surgical resection is an acceptable and adequate treatment for ectopic mediastinal meningiomas.

**Table 1:** Clinical features of reported primary mediastinal meningiomas.

authors	published	year sex	site	site (origin)	symptoms	greatest diameter	subtypes	treatment	follow up
Wilson AJ et al. [5]	1979	63 M	right	sympathetic chain (Stellate ganglion)	Horner's syndrome	4.0 cm	ND	surgery	ND
Falleni M et al. [6]	2001	45 F	right	mediastinal paravertebral lesion	none	4.5 cm	transitional	surgery	11 years disease free
Palimento D et al. [7]	2006	45 F	left	posterior paravertebral lesion	spontaneous hemothorax	12 cm	angioblastic	surgery	ND
Chen F et al. [8]	2009	41 M	right	anterior mediastinum	chest distress cough	12 cm	malignant	surgery + radiation	9 months disease free
Present case		64 M	left	anterior mediastinum	oppressing feeling	9 cm	atypical	surgery	12 months disease free

ND: Not described

## Conclusions

Here, we report an ectopic atypical meningioma arising from the anterior mediastinum because of its extreme rarity. Surgery is a feasible and effective treatment strategy for ectopic meningioma of the mediastinum.

## Consent

Written informed consent was obtained from the patient for publication of this case report and the accompanying images. A copy of the written consent is available for review by the Editor-in -Chief of this medical journal.

## List of abbreviations

CT: computed tomography; MRI: magnetic resonance imaging; H-E: hematoxylin and eosin; PAS: periodic acid-Schiff; ABC: avidin-biotin peroxidase complex; EMA: epithelial membrane antigen; αSMA: α smooth-muscle actin; hpf: high-power fields.

## Competing interests

The authors declare that they have no competing interests.

## Authors' contributions

AM drafted and co-wrote the manuscript with JH and HK. AM, TK, and EY were involved in the clinical care of the patient. JH reported pathological findings and prepared slides for manuscript inclusion. All authors have read and approved the final version of the manuscript.
